# Emergence of SARS-CoV-2 New Variants and Their Clinical Significance

**DOI:** 10.1155/2022/7336309

**Published:** 2022-05-28

**Authors:** Hitesh Singh, Nisha Dahiya, Mahima Yadav, Neelam Sehrawat

**Affiliations:** Department of Genetics, Maharshi Dayanand University, Rohtak, Haryana, India

## Abstract

COVID-19 is a respiration-related disease caused by SARS-CoV-2 and was identified in China's Wuhan city. More than 223 countries are affected by the disease worldwide. The new variants of the COVID-19 virus are causing problems, from average to life-threatening pneumonia and acute respiratory distress syndrome (ARDS). Presently, there are 170 vaccine candidates, out of which 10 have been approved by the WHO for vaccination, such as Ad26.COV2.S, Pfizer/BioNTech, COVISHIELD, Covovax, Moderna, KoviVac, and some other vaccines to combat the deadly SARS-CoV-2 infection. From all these vaccines, Pfizer/BioNTech and Moderna are showing the highest efficacy against COVID-19. These vaccines are highly efficient against COVID-19 disease, but their potentiality against new variants remains a question. COVID-19 vaccines are highly effective at preventing severe illnesses, hospitalizations, and death. The antibodies elicited by earlier infection or vaccination are the key for possible protection against SARS-CoV-2. The problem has been exacerbated by new information from Africa on the origins of the novel contagious SARS-CoV-2 strain. These new strains occur due to unique mutations in the spike protein, which modify SARS-CoV-2 transmission and infection capabilities, limiting the efficacy of the COVID-19 vaccination. Hence, there is a need to find a potential vaccine against it.

## 1. Introduction

**Figure 1:**
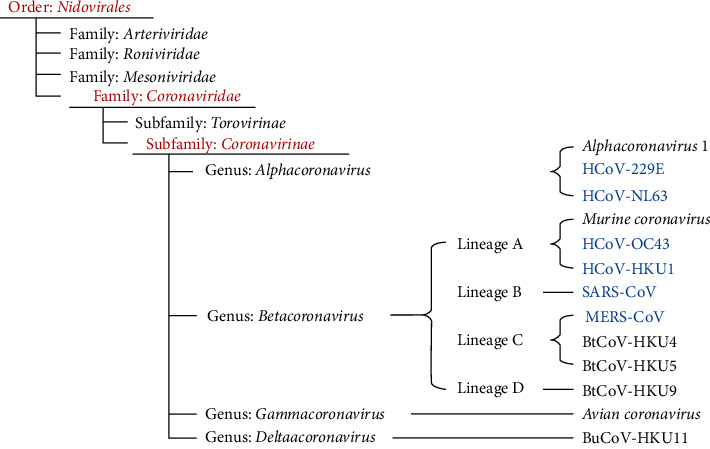
Classification of human CoV and other CoV.

The earliest human coronaviruses, OC43 and 229E, were discovered in the 1960s, followed by SARS-CoV in 2003, HCoV-NL63 in 2004, HKU1 in 2005, MERS-CoV in 2012, and ultimately the SARS-CoV-2 outbreak in December 2019 [1, 2]. In early December 2019, in China, in the city of Wuhan, some patients were diagnosed with pneumonia. Later on, it was confirmed by metagenomics analysis using next-generation sequencing of the sample of bronchoalveolar lavage of pneumonia patients and its binding ability with ACE2 receptor led the researcher to dub it as nCoV-2019 on 31 December 2019 at Wuhan Institute of Virology [3, 4]. However, due to the lower availability of ACE2, numerous coreceptors, alternative receptors, and attachment factors, including heparan sulphate, neurophilins, sialic acids, GRP78, and CD147 (BSG), have been discovered to aid virus invasion in the respiratory system [2, 5]. Based on phylogeny, taxonomy, and proven experience, nCoV-2019 was named as SARS-CoV-2 by the Coronaviridae Study Group (CSG) [6]. On 12 March 2020, the World Health Organization (WHO) declared this disease a pandemic, as it spread to other countries rapidly through human-to-human transmission [1, 2]. There is a concern on its origin that from which animals it originated and whether it can be transmitted from animals. The SARS-CoV-2 genetic sequence is 79.5% similar to SARS-CoV and 96.2% homologous to a bat coronavirus (HKU9-1) [3]. Human coronaviruses are classified as members of the Nidovirales order, which includes the families Coronaviridae, Arteriviridae, and Roniviridae, as illustrated in Figure 1. Coronaviridae is further subdivided into the Coronavirinae and Torovirinae families. Alpha, Beta, Gamma, and Delta are the four groups that make up the Coronavirinae subfamily. Mammals are infected by Alpha and Beta, while birds are infected by Gamma and Delta [2]. When it comes to phylogenetic classification, human coronaviruses are categorised as Baltimore class IV viruses since they are enveloped and contain positive-sense single-strand RNA. Till now, seven species of coronaviruses are known; these are HCoV-NL63, HCoV-229E, HCoV-OC43, HCoV-HKU1, SARS-CoV, MERS-CoV, and SARS-CoV-2 from which the first two belongs to the Alpha genus while others to Beta genus. The *β*-coronaviruses can infect animals and human beings with asymptomatic infections and lead to an outbreak as these are single-stranded RNA-enveloped viruses [7]. Bats seem to be the intermediatory host between human and bat transmission chains [34, 45]. The phylogenetic tree of the SARS-CoV-2 virus shows that it is a recombinant virus, with about 89 percent homology with the SARS bat virus, SCCoVZC21 strain (NCBI accession number MG772934), and bat-SL-CoVZC45 (NCBI accession number MG772933), and about 92–96 percent homology with RaTG13, the bat coronavirus, according to a recent study [2, 8]. The coronaviruses are polyhedral spherical viruses with a diameter of 80 to 160 nm and a large genome size of approximately 27.6–31.6 with trimers of spike proteins as projections which are visible on an electron microscope [9, 10]. The membrane glycoprotein (*M*) protects the envelope by strengthening the membrane and attaches itself to the nucleocapsid which binds to the RNA genome, and it consists of a coat protein (*E*) which helps in pathogenesis, assembly, and release of the virus [9, 11]. Coronavirus has a single-stranded positive-sense RNA genome with many open-reading frames, a cap at the 5′ terminus, and a poly(*A*) tail at the 3′ terminus. Replicase, S-E-M-N glycoproteins, and other minor ORFs representing subproteins that are distributed throughout structural genes make up the constant arrangement of genes from the 5′ to 3′ end [12, 13]. As compared to SARS-CoV, it has been found that SARS-CoV-2 has more ability to infect and replicate inside alveolar tissues with milder symptoms causing pneumonia [11, 14]. It can attach to the ACE-2 (angiotensin-converting enzyme 2 receptor), and the spike protein will mediate membrane fusion and viral entry [2, 15]. The disease is spread primarily through respiratory droplets such as coughing and sneezing when people are in proximity [2, 16, 17]. COVID-19-confirmed cases totalled 469 million on April 10, 2022, with 6 million deaths reported to the WHO. A total of 11.6 billion vaccine doses have been delivered as of April, 2022 [18]. COVID-19 instances decreased by 24 percent and mortality by 18% from 14 March 2022 to 10 April 2022 in four consecutive weeks. 7.2 million new cases and over 22,336 new deaths were reported in this week. The incidence of weekly cases decreased in all regions, with the Americas region reporting the highest increase (−4 percent), followed by South-East Asia (−8 percent), Europe (−26 percent), the Eastern Mediterranean (−4 percent), and the Western Pacific (−38 percent), and Africa (−17 percent). The death rate also decreased this week in Americas (−19%), Western Pacific (−26%), South-East Asia (−15%), Eastern Mediterranean (−18%), and European Regions (−16%), all of them reported a drop in the number of deaths [18].

The SARS-CoV-2 transmission is influenced by various abiotic factors such as climate, temperature, humidity, wind speed, air, and water quality, solid surfaces/interfaces, and frozen food, and biotic factors like age, sex, gender, blood type, population density, and behavioural characteristics. Based on transmission, persistance, and infectivity, there are various therapeutics approaches which have been employed to fight against SARS-CoV-2 [19]. There are various therapeutic approaches which have been employed to fight against SARS-CoV-2 infection. Previously, some potential antimalarial drugs like hydroxy chloroquine and azithromycin, antifilarial drug ivermectin, and antiviral drugs have been tested by many research groups worldwide for their possible effect against the COVID-19, but they did not show effective response on COVID-19 patients. The combination of these drugs such as hydroxychloroquine and ivermectin have been identified to act by creating the acidic condition in cells and inhibiting the importin (IMPa/b1)-mediated viral import [20]. Recently, mesenchymal stem cells (MSCs) have been introduced as a potential therapeutic approach for treating SARS-CoV-2 [21]. *In silico* study showed an inhibitory effect of ilimaquinone (marine sponge metabolite) against this new virus [22]. Some plant-derived natural products are also under investigation to develop a drug against this life-threatening virus. Besides all therapeutic approaches, vaccine development is the most promising therapy to control COVID-19 infection. There have been approximately 200 candidates under the clinical investigation, 69 candidates in 3^rd^ clinical trial, 69 candidates in 2^nd^ clinical trial, and 48 are in phase 1 trial. There are 10 vaccine candidates approved by the WHO (Table 1) for vaccination [18] globally, but as this virus is mutating quickly, it is creating a havoc every time.

**Figure 2:**
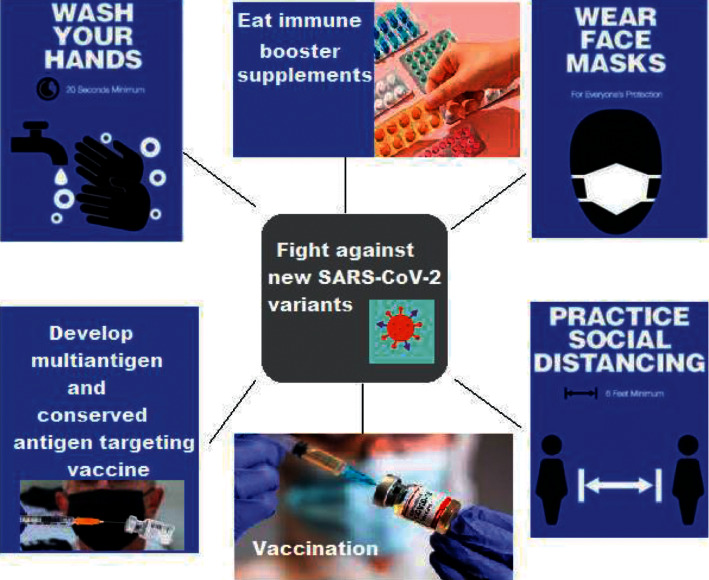
Safety precautions used to avoid COVID-19 new variants infection.

The severity of infections has grown due to a variety of SARS-CoV-2 mutations. The new and emerging respiratory virus threats advisory group (NERVTAG) in the United Kingdom published a document containing the findings of numerous preliminary analyses of B.1.1.7 [23]. In England, a highly transmissible variant with 8 mutations on the S protein was detected, out of which 3 were main antigenic determinants for vaccine licensed there [23]. In 2021, NERVTAG informed that B.1.1.7 is more infectious and severe than the parent virus. P.1 is another highly infectious variant reported in Brazil which led to collapsing of health department in the mid-2020 [23]. In 2020, a new variant B.1.351 was discovered in South Africa, and Moderna claimed that its vaccine is effective against both B.1.1.7 and B.1.351 variants based on in vitro research. The company Pfizer also claimed based on laboratory studies that their vaccine works against B.1.1.7 variant, but there is no proof as these studies were not peer-reviewed [23]. With the emergence of new variants, the severity of disease will increase, and vaccines will not work against the new variants. Therefore, for preventing disease transmission due to these new variants, we need to follow the WHO recommended control measure and develop effective vaccine (Figure 2). The world has been terrified by the SARS-CoV-2 outbreak. There is a need to understand the pathophysiology of the virus, find a solution, and in the meanwhile fight against the COVID-19 pandemic. This review attempts to give recent Information regarding COVID-19 variants to better comprehend this changing novel virus and its relevance.

## 2. The Emergence of New Variants of SARS-CoV-2

**Figure 3:**
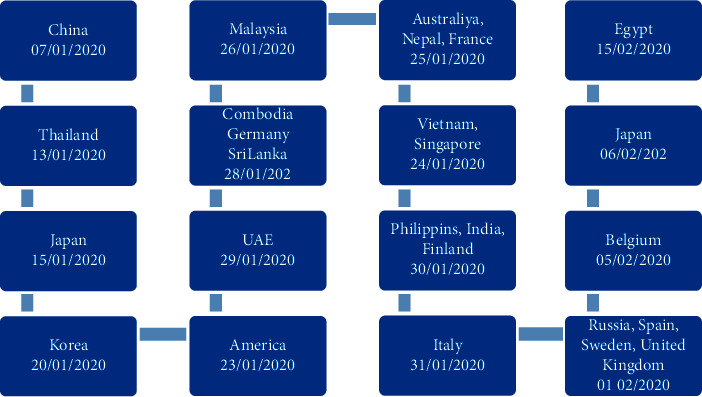
History of the origin and spread of COVID-19 infection globally [42].

The WHO received a lot of public health issues due to the SARS-CoV-2 new variants (Table 2). To combat COVID-19 infection, viral genomic sequences have been developed, and over one million SARS-CoV-2 sequences have been submitted to GISAID (https://www.cdc.gov/coronavirus/2019/variants/variant). The available genomic information could be used in tracking the outbreaks, global spread, and evolution of SARS-CoV-2. The evolution of SARS-CoV-2 has continued and emerged as new variants that have spread all over since its beginning, as shown in Figure 3.

### 2.1. Classification of SARS-CoV-2 New Variants

In the United States, epidemiological data, sequence-based genome analysis, and laboratory analysis are all used to explore the SARS-CoV-2 genome. Based on genetic substitution, the US authorities classified the SARS-CoV-2 novel variants under four classes in line with the WHO classification.

#### 2.1.1. Variant of Being Monitored

Alpha (B.1.1.7, Q.1-Q.8), Beta (B.1.351, B.1.351.2, B.1.351.3), Gamma (P.1, P.1.1, P.1.2), Epsilon (B.1.427 and B.1.429), Eta (B.1.525), Iota (B.1.526), Kappa (B.1.617.1), B.1.617.3, Mu (B.1.621, B.1.621.1), and Zeta (P.2) are the variants of being monitored.

#### 2.1.2. Variant of Interest

Epsilon (B.1.427 and B.1.429) and Zeta (P.2) are the variants of interest. The variants having mutation like variants of concern (VOCs) and limited spreads are classified as variants of interest.

#### 2.1.3. Variant of Concern

The World Health Organization (https://www.who.int/en/activities/tracking-SARS-CoV-2-variants/ (2021) and the US Centres for Disease Control and Prevention (CDC) have classified Delta (B.1.617.2 and AY.1 sublineages) and Omicron variants as variants of concern (VOCs) because they are widely spread and highly transmissible, increase the severity of disease, and reduce immunity developed by antibodies due to prior exposure or vaccination (https://www.cdc.gov/coronavirus/2019/variants/variant-info.html) (2021).

#### 2.1.4. Variant of High Consequence (VOHC)

There is no variant of high concern identified.

The CDC classified the SARS-CoV-2 new variant based on its sublineages. These sublineages are *Q* sublineages (Alpha), AY sublineages (Delta), and P.1 sublineages (Gamma).

### 2.2. SARS-CoV-2 Mutation

Currently, circulating SARS-CoV-2 variants with several mutations keep spreading in the community and replicating their existence. These variants can raise the risk of infection and viral transmission and decrease its protection by neutralising monoclonal antibodies and immunisation. Alpha, Beta, Gamma, and Delta variants are the four lineages of SARS-CoV-2 that have expanded rapidly and globally since December 2020. Most of the variants emerge from mutations related to the spike protein, and some occur in nonspike.

### 2.3. Spike Protein Mutation and Its Effect on Molecular Interaction and Lethality

According to the recent reports, the situation has been complicated due to the introduction of a new infectious strain (B.1.1.7) of SARS-CoV-2 in England [24]. Due to deletion 69–70, 144, replacements K417N, K417T, E484K, N501Y, A570D, D614G, P681H, T716I, S982A, and D1118H, and other changes in the spike protein of B.1.1.7 (deletion and substitution), the virus's infection and transmission ability have grown severely [25, 26]. The interaction and pathogenicity of the SARS-CoV-2 novel variant are altered when a mutation in the RBD domain occurs. Different variations, such as UK (N501Y), South African (K417N-E484K-N501Y), Brazilian (K417T-E484K-N501Y), and hypothetical (K417T-E484K-N501Y), have varying binding affinity to receptors, according to structural and biophysical techniques (N501Y-E484K). Variants from South Africa (K417N-E484K-N501Y) and Brazil (K417T-E484K-N501Y) have shown to be more dangerous than the UK form (N501Y) [27, 28].

### 2.4. Nonspike Mutation

Mutations other than spike proteins have been observed in novel variants, resulting in an increase in transmissibility through antagonising the host response to type I interferon's nucleocapsid D3L mutation. An example of a nonspike mutation is found in the nucleocapsid gene where an alteration in three nucleotides has been noticed. The mutation in the nonspike protein may arise due to homologous recombination in the core sequence of the nucleocapsid gene and result in a novel transcript with mutated amino acid R203K/G204R with unknown consequences [29–31].

### 2.5. Detection and Dominancy of SARS-CoV-2 New Variants

According to prior reports, new SARS-CoV-2 virus types with several mutations have been discovered in several nations, including the Kingdom of Denmark, the United Kingdom of Great Britain and Northern Ireland, and the Republic of South Africa. The epidemiological and clinical characteristics of the SARS-CoV-2 pandemic were influenced by these variations. The emergence of these new variants attracts the R&D activities of various countries to search for the impact of viral changes.

A SARS-CoV-2 variant with a D614G substitution appeared in late January or early February 2020. The D614G mutant became the dominant virus over four months in China. The animal model and human respiratory cells studies show that this D614G mutation strain has a high infectivity and transmission rate compared with the normal strain [27].

In August and September 2020, a SARS-CoV-2 with a “Cluster 5” variant identified in Denmark had a combination of mutations that were not detected earlier. This variant was among the farmed mink population and was later transmitted to the human population. Preliminary studies show that this virus neutralizes the normal strain and decreases the extent and duration of immunity developed by natural infection and vaccination. By September 2020, only 12 human cases had been identified in Denmark, and studies show that this variant is not spreading widely [27, 32].

SARS-CoV-2 VOC 202012/01 (Variation of Concern, year 2020, month 12, variant 01) was reported in the United Kingdom of Great Britain on December 14, 2020 (https://covariants.org/variants/21K). There were 23 nucleotide changes in this variation. This novel variety is the product of 23 nucleotide changes and is not phylogenetically related to the conventional SARS-CoV-2 strain. The novel virus lineage was first detected in Southeast England, where it demonstrated dominance over another virus lineage in that area as well as London. SARS-CoV-2 VOC 202012/01 has a high transmissibility rate, according to preliminary epidemiologic, modelling, phylogenetic, and clinical studies. However, there was no difference in disease severity or infection recurrence between the regular strain and another SARS-CoV-2 variant in a preliminary clinical investigation [32].

Another type of mutation was detected in the VOC 2020/12/01 variant due to the deletion at position 69/70. It affects the performance of PCR assays for COVID-19 infection diagnosis targeting the S gene. The other variants do not seem to have any significant impact on diagnostic methods like PCR-based detection or antigen-based assays. VOC-202012/01 has been recorded in 31 different countries/territories/areas in five of the six WHO regions as of December 30, 2021.

On 18th December 2020, the South African government announced the discovery of a new variety 501Y.V2 with the N501Y mutation [27]. The N501Y mutation is also seen in the SARS-CoV-2 VOC 202012/01 variant found in the UK, although evolutionary study reveals that these two variants are not related. This 501Y.V2 strain has a high transmission rate, according to the South African Health Ministry, and it has replaced earlier SARS-CoV-2 viruses circulating in the Eastern Cape, Western Cape, and KwaZulu-Natal provinces. The 501.V2 variety is quickly replacing another virus lineage in South Africa, according to genomic analysis. But there is a lack of evidence about the severity of the new variants. There is a need for further investigation of this variant's impact on the transmission rate and infection severity. As on 30 December 2020, the 501Y.V2 South African variant has been reported in four other countries [32]. A new variant 478K.V1 was detected on 11 May 2021 in India. Now, the researchers hypothesized that it might be responsible for the third wave of corona infection and unvaccinated people provide the host for infection and transmission [27, 32].

In Brazil, two dominated lineages B.1.1.28 and B.1.1.33 were reported to be the reason of the epidemic [33]. Lineages P.1 and P.2 have mutations in the RBD domain of the spike protein. The two variants were aroused as a result of mutation B.1.1.28. lineage. Lineage P.1 with multiple mutations (L18F, T20N, P26S, D138Y, R190S, K417T, E484K, N501Y, H655Y, and T1027I) in the S protein is considered as a variant of concern (VOC) as it is responsible for second wave in the Amazonas state [27, 34, 35]. The P.2 lineage single mutation spike protein S : E484K and other mutations were located outside the S protein. On 1 March 2021, P.2 lineage was considered as a variant of interest (VOI) [36]. Between November 2020 and February 2021, a variant of interest with mutation S:E484K in lineage B.1.1.3.3 was reported in Brazil. It had four mutations in the N.9 lineage (NSP3:A1711V, NSP6:F36L, S:E484K, and NS7b:E33A). The VOI N.9 first appeared in August 2020, and it quickly expanded over Brazil's Southeast, South, North, and Northeast regions [27, 28, 37].

On 24 November 2021, the WHO reported a new variant Omicron in Botswana and South Africa. On 26 November 2021, it was categorised as a variation of concern (VOC). According to GISAID, 58 countries shared 4992 omicron sequences as of December 22. A genome study indicated that Omicron had a higher frequency of mutations than other VOCs. In comparison to the Delta variation, which has 16 mutations, Omicron has roughly 32 mutations in the spike protein, and these mutations affect the performance of PCR assays for COVID-19 infection diagnosis targeting the S gene. It also has a mutation in viral replication-related proteins including NSP12 and NSP14.2 [32, 38].

There are various theories postulated regarding the Omicron emergence: (1) circulation of infection in patients, (2) unreported new variants, (3) spike protein has improved its affinity for the ACE-2 receptor due to a mutation, (4) the reason behind a large number of mutations can be due to the involvement of unknown animal host, and (5) the low immunisation rate in low African countries has resulted in the evolution of the Omicron variant [39]. Omicron is phylogenetically related to the Gamma (P.1) variant. In the S protein, mutations like H69-, V70-, G142-, V143, Y144-, and N211- occurred and deletion 69/70 affected the binding affinity of antibodies to the S-protein [40, 41]. The spike protein also has other mutations like A67V, T95I, Y145D, G339D, S371L, S373P, S375F, K417N, N440K, G446S, S477N, T478K, E484A, Q493R, G496S, Q498R, N501Y, Y505H, T547K, D614G, H655Y, N679K, P681H, N764K, D796Y, N856K, Q954H, N969K, and L981F. The mutation in the S1-S2 furin cleavage site at H655Y, N679K, and P681H has increased the transmission ability of the variant [38]. Several mutations were also detected in ORF1a, ORF1b, ORF9b, the envelope gene, and the matrix gene. The envelope gene has only one mutation at T9I, but the matrix gene has D3G, Q19E, and A63T 10 alterations [41] (https://www.cdc.gov/coronavirus/2019-/science/science-briefs/scientific-brief-omicron-variant.html2021) (https://covariants.org/variants/21K).

As the data show that the novel coronavirus is mutating quickly and till now there is no effective measure to control this pandemic, the contagious strains are creating havoc and a challenge for the medical facilities and economy and to the life of people. So, the best ways to control this are diagnosis at early stage, quarantine, personal hygiene, using protective mask or face shield, avoiding crowded places, and timely availability of information about pandemics to avoid stress. This comprehensive review focuses on the present mutants of SARS-CoV-2 so that the knowledge gaps can be filled and a target can be made for developing a vaccine that can fully inhibit the spread of variants.

## 3. Future Prospective

The knowledge about emergence of new variants helps to predict the future upcoming variants and their impact on transmission and severity of the infection. Most of the SARS-CoV-2 variants are due to spike mutations. Based on deep study on spike mutations and some other mutations with respect to their functional consequences, it can help to predict the amino acid sites within spikes which are more susceptible to evolve. The genome surveillance and tracking of these new variants would be important for early identification of potential variants of concern and actionable interventions.

## 4. Conclusion

SARS-CoV-2 new variants are emerged with novel epidemiological and biological characteristics. It is an RNA virus and can become more prone to mutation with a new host, resulting in the emergence of multivariants with different characteristics from their ancestors. These mutations affect the transmission rate and severity of the infection. They also prevent the diagnostic tests from detecting the virus and reduce susceptibility to antiviral and monoclonal antibodies. These novel variations have the ability to reinfect people who have recovered and have been vaccinated.

Furthermore, the currently available vaccines do not provide complete protective immunity against infection and these vaccines are less effective against variants of SARS-CoV-2. Accordingly, the public and health systems need to plan for the possibility that COVID-19 will persist and become a persistent seasonal disease. To avoid illness transmission, it is recommended to keep a safe distance, use masks and gloves, and wash hands with sanitizer or soap [19]. When dealing with the outbreak and managing patients, the SARS-CoV-2 pandemic has put pressure on the world to work together to better understand the virus's essence and seek solutions. Genomic variation surveillance can play an important role in the early identification of new variants and their related consequences. To overcome the burden of these new variants, we need to follow the WHO precautionary guidelines and get vaccinated to avoid mutations in the virus genome. The scientific community needs to develop a multiantigen vaccine and target these new variants.

## Figures and Tables

**Table 1:** The WHO EUL/PQ-approved vaccine for vaccination and its efficacy.

Manufacturer	Name of the vaccine	NRA of record	Platform	Efficacy	Approved schedule for doses	Number of countries approved	Numer of trials/no. of countries
Pfizer	BNT162b2/COMIRNATY Tozinameran (INN)	EMA	Nucleoside-modified mNRA	94%	Two doses, 21–28 days apart	141	70/26
Oxford/AstraZeneca	AZD1222	Core: EMA Non-COVAX	Recombinant ChAdOx1 adenoviral vector encoding the spike protein antigen of SARS-CoV-2	70%	Two doses, 4–12 weeks apart	138	62/30
Bharat Biotech	Covaxin	CTRI	Inactivated, produced in Vero cells	70–90%	Two doses, 28 days apart	14	10/2
Serum Institute of India	Covishield (ChAdOx1_nCoV-19)	DCGI	Recombinant ChAdOx1 adenoviral vector encoding the spike protein antigen of SARS-CoV-2	70%	Two doses, 4–12 weeks apart	47	2/1
Sinopharm/BIBP (Beijing Bio-Institute of Biological Products Co-Ltd)	SARS-CoV-2 Vaccine (Vero Cell), Inactivated (lnCoV)	NMPA	Inactivated, produced in Vero cells	79–86%	Two doses, 21–28 days apart	90	25/12
Sinovac	SARS-CoV-2 Vaccine (Vero Cell), Inactivated	NMPA	Inactivated, produced in Vero cells	50%	Two doses, 14–28 days apart	54	37/9
Moderna	Spikevax	EMA	mNRA-based vaccine encapsulated in lipid nanoparticle (LNP)	95%	Two doses, 28 days apart	83	56/22
Janseen (Johnson & Johnson)	Ad26.COV2.S	EMA	Nonreplicated viral vector recombinant, replication-incompetent adenovirus type 26 (Ad26) vectored vaccine encoding the (SARS-CoV-2) Spike (S) protein	66%	One dose	108	20/22
Covovax	Serum Institute of India	EMA	Recombinant protein	80–95%	Two doses, 21–28 days apart	3	2/1
Nuvaxovid	(Novavax)	EMA	Recombinant protein	80–95%	Two doses, 21–28 days apart	36	15/12

**Table 2:** SARS-CoV-2 new variants and its emergence.

Variant	Lineage	First documented samples
Alpha	B.1.1.7	UK (Sep 2020
Beta	B.1.351	South Africa (May 2020)
Gamma	P.1	Brazil (Nov 2020)
Delta	B.1.617.2	India (Oct 2020)
Omicron	B.1.1.529	South Africa (23 Nov 2021)
Epsilon	B.1.427 and B.1.429	USA (March 2020)
Zeta	P.2	Brazil (Apr 2020)
Eta and Iota	B.1.525 and B.1.526	Multiple (Dec 2020)
Theta	P.3	Philippines (Jan 2021)
Kappa	B.1.617.1	USA (Nov 2020)
Lambda	C.37	India (Oct 2020)
Mu	B.1.621	Peru (Aug 2020)

## Data Availability

No data were used to support this study.
